# Human Chorionic Gonadotropin: The Pregnancy Hormone and More

**DOI:** 10.3390/ijms18051059

**Published:** 2017-05-14

**Authors:** Charalampos Theofanakis, Petros Drakakis, Alexandros Besharat, Dimitrios Loutradis

**Affiliations:** IVF Unit, 1st Department of Obstetrics & Gynecology, National & Kapodistrian University of Athens, Alexandra Hospital, 11528 Athens, Greece; charalampostheofanakis@yahoo.com (C.T.); pdrakakis@hotmail.com (P.D.); drabesharat@gmail.com (A.B.)

**Keywords:** hCG, LH/CGR, ovulation induction, oocyte maturation, endometrial synchrony, immunization

## Abstract

To thoroughly review the uses of human chorionic gonadotropin (hCG) related to the process of reproduction and also assess new, non-traditional theories. Review of the international literature and research studies. hCG and its receptor, LH/CGR, are expressed in numerous sites of the reproductive tract, both in gonadal and extra-goanadal tissues, promoting oocyte maturation, fertilization, implantation and early embryo development. Moreover, hCG seems to have a potential role as an anti-rejection agent in solid organ transplantation. Future research needs to focus extensively on the functions of hCG and its receptor LH/CGR, in an effort to reveal known, as well as unknown clinical potentials.

## 1. Introduction

Human chorionic gonadotropin (hCG), also known as “the hormone of pregnancy” has an important role in human reproduction. Several studies have demonstrated its crucial part in establishing and maintaining pregnancy, through placentation and early embryo development. In addition to that, hCG has been used in assisted reproduction protocols by mimicking the endogenous Luteinizing Hormone (LH)-surge during late follicular phase to induce final follicle and oocyte maturation and ovulation. Recent data suggest a participation of regular hCG and its variant, hyperglycosylated hCG in hemochorial placentation, which is important for the development of the advanced human brain. Moreover, it has been proposed that hCG could act as an anti-rejection agent during solid organ transplantation, a concept that is based on the inhibition of maternal immunological response to the invading blastocyst. 

## 2. hCG and Assisted Reproduction

Controlled ovarian stimulation (COS) for in vitro fertilization (IVF) is based on various protocols with different ovarian response amongst women with issues of infertility. IVF experts face multiple failed attempts in every day practice, the goal being to try and find the ideal therapeutic approach for each patient [1]. The use of gonadotropins and the variety of expression of its receptors in each patient have been at the top of the research pyramid for many years [2].

We know by now that LH is involved in follicle maturation, beginning from the antral stage. Primordial and primary preantral are considered gonadotropin independent stages of follicular development, since cumulus and theca cells lack FSH and LH receptors. However, studies have demonstrated the presence of FSH and LH receptors from the secondary preantral and from the antral stage onwards, respectively [3]. LH receptors are also present in the theca cells from the secondary preantral stage onwards, while they are lacking FSH receptors. Gonadotropin receptor allocation in follicular cells expresses the two-cell two-gonadotropin theory [4]. According to basic knowledge, LH stimulates androgen production by the theca cells, while FSH promotes aromatase enzyme activity and thus the utilization of androgens as a substrate for estrogen biosynthesis.

FSH regulates recruitment, selection and dominance of ovarian follicles, while LH promotes final maturation and ovulation, making the two gonadotropins act synergistically in the process of follicular growth [5,6]. Even though the preantral stage can be reached in the absence of LH, it is an important factor in both oocyte and follicular cells development through modification of the steroid and protein micro- and macro-environment [5,7]. These physiologic changes have a prominent role in oocyte maturation, ovulation and subsequent fertilization and implantation [8]. Studies on non-human primates have shown that LH may act by increasing intra-ovarian androgens, which in turn promote FSH responsiveness in granulosa cells [9].

hCG is being used in assisted reproduction protocols to mimic the mid-cycle LH surge because of the degree of homology between the two hormones [10]. hCG is characterized by structural similarities with LH, while they share the same receptor, LH/CGR. The factor that distinguishes hCG is the longer half-life of 36 h [11] while the elimination half-life of recombinant LH is estimated to be about 10–12 h [12]. The slower plasma metabolic clearance of hCG consists of a rapid phase in the first 5–9 h following intramuscular (IM) administration and a slower phase in the first 1–1.3 days after administration. Apart from that, hCG demonstrates a stronger LH/CGR receptor binding affinity, probably due to differences in the carbohydrate moiety, which may make the molecule more sensitive to the binding receptor [13] and is much more potent than LH [14].

LH and hCG have the same α-subunits and a high cysteine content and also present with an identical natural function; inducing ovulation and supporting lutein cells. Their main differences involve the sequence of the β-subunit, the regulation of the secretion of the two hormones, the carbohydrate component and the pharmacokinetics of clearance of hCG as opposed to LH [15,16].

The LH/CGR has an almost ubiquitous distribution in reproductive tissues, a fact that may suggest that the actions of hCG might be more extensive than once thought to be. Although it is mainly located in the gonads, ovary and testis, it can also be found in extra-gonadal reproductive organs, such as the uterus and the fallopian tubes. Moreover, studies have stated that non-reproductive tissues such as the skin, breast, adrenals, thyroid, neural retina and neuroendocrine cells express LH/CGR too [17]. Regardless of the administration or not of FSH, low-dose hCG can support the development and maturation of larger ovarian follicles that have acquired granulosa cell LH/CGRs, possibly providing effective and safer ovulation induction regimens. In a recent study [18], it was demonstrated that the addition of hCG to rFSH in a short GnRH-agonist protocol throughout the early follicular phase had a beneficial effect in terms of pregnancy rates, while hCG was also associated with better quality embryos [18]. A possible explanation would be the direct induction of theca cells androgen production, which is subsequently transformed to estrogen in granulosa cells through an increased aromatization rate.

In a previous study by our group, it was demonstrated that infertile women pre-treated with hCG seemed to have higher E2 levels on the day of hCG administration for triggering ovulation and resumption of meiotic division, which is related to better quality embryos and increased pregnancy rates [19].

In a prospective randomized study by Drakakis et al. [13], the authors tried to determine whether low dose hCG added to rFSH for ovarian stimulation could produce better results compared to the addition of rLH in women entering IVF-ET in a short protocol, especially in those women with previous failed IVF attempts. Results showed that, due to the use of hCG, less gonadotropin ampoules were needed for ovarian stimulation and higher fertilization and higher pregnancy rates were recorded. There was also a tendency for a better implantation rate, even in women of advanced reproductive age with higher basal FSH levels, which are often considered to have poor ovarian response to stimulation. In addition, the percentage of mature oocytes and the number and quality of embryos was comparable between rLH and hCG groups. This could state that hCG, in the specific dose and way of administration, had no drawback effect on ovarian stimulation.

A possible explanation provided by the authors could be that the longer plasma half-life of hCG and its greater potency (roughly six to eight times greater than that of LH) leads to highly effective and more stable occupancy of the LH/CG receptors. Given the fact that serum E2 levels in patients who received rLH were significantly lower than in patients treated with hCG, the occupation of the LH/CG receptor in the rLH-administered patients is less than to the hCG stimulated patients. Moreover, it was demonstrated that mRNA levels of the LH/CG receptor after ovarian stimulation were significantly higher among women receiving hCG, compared to those receiving rLH [13].

Assessing the use of recombinant LH and hCG in oocyte maturation during clinical In Vitro Maturation (IVM), a number of studies have investigated the differences between the effects of these gonadotropins in a well-designed in vitro system. In the absence of the use of FSH, low-dose hCG can support the development and maturation of larger ovarian follicles (≥15 mm in diameter) that have acquired granulosa cell LH/CGRs while, at the same time, it inhibits the demise of smaller follicles lacking these receptors, thus being dependent on FSH stimulation [14,20,21]. Dinopoulou et al., studied the effect of recombinant-LH and hCG in the absence of FSH on IVM, fertilization and early embryonic development of mouse germinal vesicle (GV)-stage oocytes. The LH/hCG receptor was expressed in all stages of in vitro matured mouse oocytes and in every stage of early embryonic development. Furthermore, the addition of hCG in IVM cultures of mouse GV oocytes significantly increased the recorded maturation rates [22].

In search of another potential use of hCG, Filicori et al., studied the possibility of replacing FSH with low-dose hCG in order to complete controlled ovarian stimulation. The authors included infertile patients, separated in two study groups. In the first group, patients received recombinant FSH of hMG throughout COH, while in the second group ovarian priming was induced with recombinant FSH/hMG followed by a low dose of hCG. The authors concluded that low-dose hCG in the late COH stages reduced recombinant FSH/hMG administration while ICSI outcome had no significant difference. It was also stated that follicle growth and maturation were stimulated regardless of FSH dosage and the protocol led to a reduced number of preovulatory follicles. Moreover, there was no premature luteinization and a more estrogenic intrafollicular environment was recorded in the low-dose hCG group [23]. The same group also tested the effect of hCG on patients with hypogonadotropic hypogonadism. They concluded that the administration of highly purified FSH and low-dose hCG (50 IU/day) led to an acceleration of follicular development in follicles >14 mm and a reduction in the dose of FSH requirement, while the duration of COS was also shortened [24]. 

## 3. hCG and Endometrial Synchrony

We have already known for more than two decades that LH/CG receptors are expressed in the human uterus [25]. These receptors are found in multiple cells types, especially in epithelial cells [26]. Based on this knowledge, we can hypothesize that hCG could lead to the improvement of uterine receptivity via the enhancement of endometrial quality and stromal fibroblast function. Moreover, through its actions on insulin-like growth factor binding protein-1 (IGFBP-1) and vascular endothelial growth factor (VEGF), hCG was found to stimulate endometrial angiogenesis and growth, thus extending the implantation window and increasing pregnancy rates [14,27]. IGFBP-1 is selectively produced by decidualized endometrial stromal cells and represents a marker of decidua formation and its concentrations are cycle-dependent. One study has shown that women investigated before day 10 after the LH surge were IGFBP-1 negative, while all women examined at day 10 or later presented with high intrauterine IGFBP-1 levels [28]. This could serve as a marker that indicates the ending of the window of implantation, leading to the hypothesis that the increase of IGFBP-1 could be functionally involved in the restriction of endometrial receptivity [14].

Intrauterine hCG infusion seems to be associated with endometrial synchrony and reprogramming of stromal development following ovarian stimulation [29]. A study by Mansour et al., demonstrated that the administration of 500 IU of hCG to the endometrial cavity prior to embryo transfer on day 3 post-oocyte retrieval lead to statistically significant higher pregnancy and implantation rates compared to the control group [30]. Pre-treatment with hCG seems to have a beneficial effect on endometrial quality, defined as endometrial thickness >8 mm and assessed by ultrasound scan on the day of egg collection [19].

Besides increasing production of hCG by the trophoblastic tissue soon after implantation, this hormone is also produced by the blastocyst and may contribute in a paracrine manner to the implantation process [31]. Studies have shown that hCG represents the first known human-embryo derived signal in maternal-fetal communication, through which the embryo influences the immunologic tolerance and angiogenesis at the maternal-fetal interface [32].

## 4. hCG and Placentation

Recent research has focused on the connection of hCG to the evolution of human hemochorial placentation. Several studies have shown that the various forms of hCG were discovered with the appearance of hemochorial placentation [33,34,35]. This communication system between the mother and the fetus is essential for the development of larger brains in humans and advanced primates [36]. The presence of LH/CG receptors on spiral arteries in the myometrium and decidua and the role of hCG in the angiogenesis of these spiral arteries has been known for more than a decade [37,38,39]. Using Doppler to study spiral arteries following in vivo hCG injections, Toth et al., demonstrated that hCG decreases vascular resistance in the vessels, causing dilation and promoting blood flow [40]. A significantly larger variant of regular hCG, hyperglycosylated hCG is believed to play an important role in the establishment of hemochorial placentation. Hyperglycosylated hCG is produced by the invasive extravillus cytotrophoblast cells of the placenta in humans [41,42,43]. It seems that its role lies in promoting invasion during placentation, while, on the other hand, it acts as an autocrine factor blocking apoptosis of these cytotrophoblastic cells [44]. Based on these facts, Cole et al., suggested that hyperglycosylated hCG is responsible for the invasion of cytotrophoblast cells to the uterus and achieving an implantation that is as deep as possible, while regular hCG enhances spiral artery growth and multiplicity to meet and provide nutrition to the invading villi [45].

It is established knowledge that invasion distinguishes hemochorial placentation from more primitive placental models. However, obstetric complications are unique to humans, compared to other primates. Humans have developed a more sophisticated and complex model of placentation, in order to support the more demanding human brain. Moreover, it is estimated that nearly 41% of pregnancies in humans lead to miscarriages, early pregnancy losses and biochemical pregnancies, while this percentage declines to lower than 10% in most mammalian species [46]. Sasaki et al., suggested that most pregnancy failures are due to inadequate hyperglycosylated hCG production on the day of implantation of the blastocyst [47]. Researchers have also demonstrated that pregnancy-induced hypertension, preeclampsia and eclampsia are complications of incomplete hemochorial placentation mechanisms, which usually take place at the end of the first trimester of pregnancy [48,49]. The possible association between hCG and hyperglycosylated hCG with hemochorial placentation could lead to new cures and treatments for choriocarcinoma and invasive mole [45].

## 5. hCG as an Anti-Rejection Agent

Based on the existence of a unique relationship between the mother and the fetus and given the important role of hCG in the establishment of this relationship, a new theory has been developed, which indicates that hCG could be the answer in the chronic rejection in solid organ transplantation. hCG promotes tolerance through a number of actions on the human immune system [32,50], while recently, it was found that hCG prolongs skin allografts in mice. Moreover, women receiving hCG preconditioning prior to IVF had reduced inflammatory IL17 but increased anti-inflammatory IL27 and IL10 [51]. In addition to this effect, the improvement in the symptoms of rheumatoid arthritis during pregnancy is due in part to the hCG induced shift of Th1 mediated cellular immunity to a pro-pregnancy Th2 immunity and an increase in T regulatory cell function. Therefore, it seems that these changes are in favor of both pregnancy and reduction in pathogenic RA immune activity [52,53]. hCG has also been used with success in the management of paraneoplastic neuropathy mediated by anti-Hu antibodies [54].

Given the fact that the female organism enters a better state when pregnant, and women with minimally aggressive trophoblastic neoplasia and hCG levels over 3000 mIU/ml remain generally in good health [55], it is a safe hypothesis that the use of hCG comes without any side effects and it is both subtle and specific in its action. This is an important difference between hCG and the use of the current anti-rejection agents, such as corticosteroids, ciclosporin, tacrolimus azathioprine, mycophenolate mofetil and a range of T-cell specific antibodies [56]. Moreover, hCG could be used as a therapeutic agent against autoimmune diseases such as rheumatoid arthritis and Sjögren’s syndrome. Being cheap and easy to use with minimum adverse effects, further studies need to be made in order to assess the efficacy of hCG on the treatment of autoimmune disorders [57].

## 6. Conclusions

Normal pregnancy is nature’s way of teaching us that there could be an alternative path to reduce or even prevent graft rejection, based on the effect of hCG on immune mechanisms of allo-recognition during implantation and embryo development. Apart from the positive effects of hCG on ovulation induction during controlled ovarian stimulation protocols and on endometrium receptivity during implantation, research on solid organ transplantation remain to be thoroughly tested in the future. We hereby state that understanding the physiologic roles of hCG is of outmost importance, in order to fully take advantage of its therapeutic potentials. It seems that this “hormone of pregnancy” has more treatment applications than once believed and one can only hope that future research will reveal even more promising uses of this old-new hormone.
